# Irigenin inhibits glioblastoma progression through suppressing YAP/β-catenin signaling

**DOI:** 10.3389/fphar.2022.1027577

**Published:** 2022-11-30

**Authors:** Jiayun Xu, Shanshan Sun, Wei Zhang, Jianhong Dong, Changgang Huang, Xin Wang, Mengxian Jia, Hao Yang, Yongjie Wang, Yuanyuan Jiang, Liying Cao, Zhihui Huang

**Affiliations:** ^1^ College of Pharmacy, Hangzhou Normal University, Hangzhou, Zhejiang, China; ^2^ Key Laboratory of Elemene Class Anti-Cancer Chinese Medicines, Engineering Laboratory of Development and Application of Traditional Chinese Medicines, Collaborative Innovation Center of Traditional Chinese Medicines of Zhejiang Province, Hangzhou Normal University, Hangzhou, Zhejiang, China; ^3^ Laboratory of Aging and Cancer Biology of Zhejiang Province, School of Basic Medical Sciences, Hangzhou Normal University, Hangzhou, China

**Keywords:** irigenin, glioblastoma, proliferation, migration, apoptosis, cell cycle arrest, YAP, β-catenin

## Abstract

Glioblastoma (GBM) is the most malignant glioma in brain tumors with low survival and high recurrence rate. Irigenin, as an isoflavone compound extracted from Shegan, has shown many pharmacological functions such as antioxidant, anti-inflammatory and anti-tumor. However, the effects of irigenin on GBM cells and the related molecular mechanisms remain unexplored. In this study, we found that irigenin inhibited the proliferation of GBM cells in a dose-dependent manner by several assays *in vitro*. Subsequently, we found that irigenin arrested cell cycle at G2/M phase and induced apoptosis of GBM cells *in vitro*. In addition, irigenin inhibited the migration of GBM cells. Mechanically, we found that irigenin treatment decreased the expression of YAP (yes-associated protein), suppressed β-catenin signaling. Furthermore, overexpression of YAP partially restored the anti-tumor effects of irigenin on GBM cells *in vitro*. Finally, we found that irigenin inhibited the growth of tumor in GBM xenograft mice model through inactivation of YAP. Taken together, these results suggest that irigenin exerts its anticancer effects on GBM *via* inhibiting YAP/β-catenin signaling, which may provide a new strategy for the treatment of GBM.

## 1 Introduction

Glioblastoma (GBM), the most common primary malignant tumor in the brain, is extremely aggressive and in various forms with a dismal prognosis, and most patients die within 1 year after diagnosis (Miller and Perry, 2007). Primary GBM may reappear from the beginning without clinical, radiological or histopathological evidence of a less malignant precursor lesion, generally affecting the elderly. In contrast, secondary GBM may develop from a low-grade tumor and develop over time, usually in young patients (Ohgaki and Kleihues, 2013). At present, the understanding of GBM is still very limited. Treatments are also limited due to blood-brain barrier (BBB) and suppression of the immune system. A combination of multiple methods including surgery, radiation and chemotherapy usually required for the patients, even so, without much benefit. TMZ, as a first-line chemotherapy drug for GBM, which is always found to be resistant after treatment, and the high recurrence rate also makes the curative effect very limited (Stupp et al., 2005; Fernandes et al., 2017; Osuka and Van Meir, 2017). Therefore, exploring drugs with good efficacy and low toxicity to treat GBM will be urgent to improve patients’ survival and prognosis.

Belamcanda chinensis (L.) is a common Chinese medicinal material. Isoflavone compounds, including irigenin, are important medicinal ingredients in this plant (Ito et al., 2001). Current studies have revealed that irigenin not only shows the effects of antioxidative and anti-inflammatory, but also has anti-tumor activity (Woźniak and Matkowski, 2015; Guo et al., 2020) including inhibition of cancer cell metastasis (Amin et al., 2016), promotion of cell apoptosis (Xu et al., 2018), cell cycle arrest (Xu et al., 2021), autophagy (Zhan et al., 2021) and extracellular matrix degradation (Zhang et al., 2021). These antitumor effects of irigenin are associated with the modulation of various signaling pathways. For example, irigenin has been proved to inhibit the development of human colon cancer by suppressing of ERK/MAPK signal pathway (Zhan et al., 2021). Similar anti-cancer effects focused on apoptosis in the most of these studies (Xu et al., 2018; Xu et al., 2021). However, the function of irigenin in GBM remains unclear.

In mammals, the hippo pathway is a kinase cascade reaction from Mst1/2 to YAP and its paralog TAZ. Dysregulation of the hippo pathway has been observed in various cancers. Yes-associated protein (YAP), as a co-activator of TEAD/TEF family transcription factors, is a highly conserved component of the hippo pathway, induces gene expression, oncogenic transformation and epithelial-mesenchymal transition, and regulates cell proliferation, apoptosis and organ growth (Harvey et al., 2013; Yu et al., 2015). There is considerable evidence showing that YAP plays an important role in malignant tumor formation and all cancers have YAP^on^ or YAP^off^, where the cells growing against the wall are YAP^on^ and the cells floating are YAP^off^ (Zhao et al., 2021). In addition, hyperactivation of YAP promotes glioma growth with a worse prognosis (Orr et al., 2011; Zhang et al., 2018; Pearson et al., 2021). However, it remains unclear whether irigenin affects the growth of GBM through YAP signaling.

In this study, we focused on the effects of irigenin on GBM cells and the underlying signaling pathway, and found that irigenin inhibited the proliferation and migration of GBM cells, caused cell cycle arrest at G2/M phase, and induced apoptosis of GBM cells, and finally found that irigenin inhibited the progression of GBM both *in vitro* and *in vivo* through suppressing YAP/β-catenin pathway. These results suggest that irigenin might be an effective therapeutic agent and YAP/β-catenin pathway could be a potential therapeutic target for the treatment of GBM.

## 2 Results

### 2.1 Anti-proliferative activity of irigenin in glioblastoma cells

**FIGURE 1:**
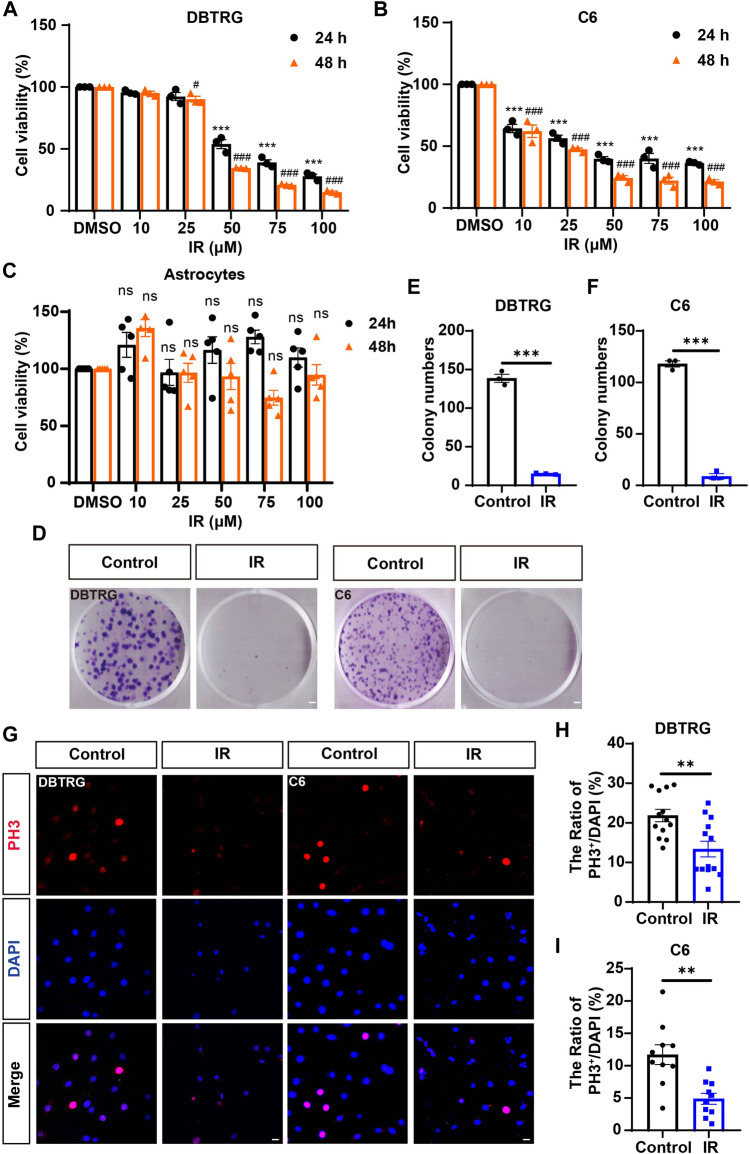
Irigenin inhibited the proliferation of GBM cells. **(A–C)** The IC_50_ of irigenin (IR) was measured by CCK-8 in DBTRG cells **(A)**, C6 cells **(B)** and astrocytes **(C)** (*n* = 3, per group, normalized to the control group). The DBTRG cells, C6 cells and astrocytes were seeded in 96-well plates at 4×10^3^/well and treated with different concentrations (0, 10, 25, 50, 75, 100 μM) for 24 h or 48 h for CCK-8 assay. **(D)** The Colony formation assay showed the anti-proliferative activity of 50 μM IR in DBTRG and C6 cells. **(E–F)** Quantification of the clone numbers in DBTRG cells **(D)** and C6 cells **(E)**, as shown in **(C)** (*n* = 3, per group). **(G)** Immunofluorescent staining of PH3 (red) in DBTRG and C6 cells treated with 40 μM IR for 24 h **(H–I)** Quantitative analysis of the percentage of PH3^+^ cells over total DBTRG cells **(H)** and C6 cells **(I)** in one field shown in **(G)** (*n* = 13, per group). Scale bar, 2 mm in figure **(D)** and 20 μm in figure **(G)**. Data were shown mean ± SEM. ^*^ *p < 0.05*, ^**^ *p < 0.01*, ^***^ *p < 0.001*, compared with control group. ^#^ *p < 0.05*, ^###^ *p < 0.001*, compared with 48 h control group.

To observe the cytotoxic and inhibitory effects of irigenin on GBM cells, DBTRG and C6 cells (GBM cell lines) were treated with different concentrations of irigenin for 24 h and 48 h respectively. The CCK-8 assay showed that irigenin reduced cell viability in a time- and dose-dependent manner with strong anti-proliferative effects of GBM cells. Irigenin effectively inhibited the proliferation of GBM cells with IC_50_ at about 50 μM (Figures 1A,B). Meanwhile, as shown in Figure 1C, CCK-8 assay showed that irigenin has no significant toxic effect on astrocytes, suggesting that irigenin might have specific toxic effects on GBM cell, but not the noncancerous normal cells such as astrocytes. We next performed a two-dimensional colony formation assay in both cell lines (Figure 1D). As expected, irigenin significantly decreased the colony numbers of DBTRG and C6 cells, compared with that in the control treatment (Figures 1E,F). Furthermore, to further confirm the anti-proliferative effect of irigenin on DBTRG and C6 cells, phosphorylated histone H3 (Phospho-Histone 3, PH3) staining, which is marked in cells during mitosis, was used to detect the proliferation rate of cells. The results showed that irigenin significantly reduced the percentage of PH3^+^ cells in both DBTRG and C6 cells treated with irigenin (Figures 1G–I). Taken together, these results suggested that irigenin inhibits the proliferative activity of GBM cells.

### 2.2 Irigenin triggered glioblastoma cell cycle arrest at G2/M

**FIGURE 2:**
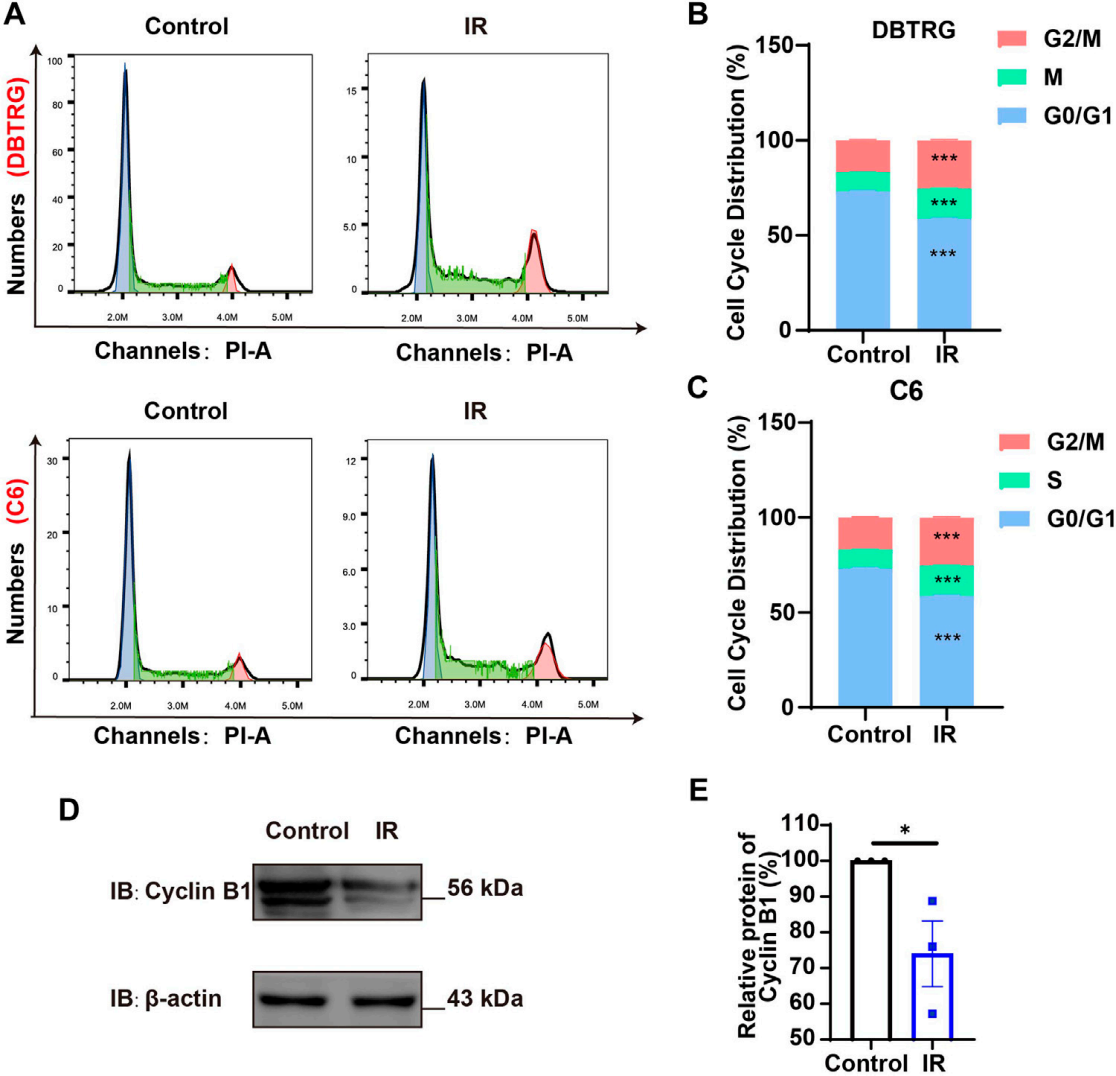
Irigenin promoted cell cycle arrest at G2/M of GBM cells. **(A)** Flow cytometry assay with PI staining showed the percentage of each cell phase in DBTRG and C6 cells treated with 50 μM IR for 24 h **(B–C)** Quantification of cell cycle percentage in DBTRG cells **(B)** and C6 cells **(C)** as shown in **(A)** (*n* = 3, per group). **(D)** Western blot detected the expression of Cyclin B1 in DBTRG cells treated with 50 μM IR. **(E)** Quantification of the relative CyclinB1 level as shown in **(D)** (*n* = 3, per group, normalized to control). Data were shown as mean ± SEM. ^*^ *p < 0.05*, ^***^ *p < 0.001*, compared with control treatment.

To further determine whether the growth inhibition of irigenin is related to cell cycle arrest, flow cytometric assay was performed. As shown in Figures 2A–C, the proportion of GBM cells treated with irigenin significantly increased at G2/M phase, compared with the control group. It is known that Cyclin B1 activates and forms a complex with CDKs, also known as CDC2 (cell division cycle), which promotes the transition of cells to the G2/M phase (Park et al., 2000). As expected, cell cycle G2/M phase-related proteins, such as Cyclin B1 was markedly decreased (Figures 2D,E, Supplementary Figure S2A–B). Taken together, these results suggested that irigenin treatment may inhibit the proliferation of GBM cells by inducing G2/M cell cycle arrest.

### 2.3 Irigenin induced apoptosis of glioblastoma cells

**FIGURE 3:**
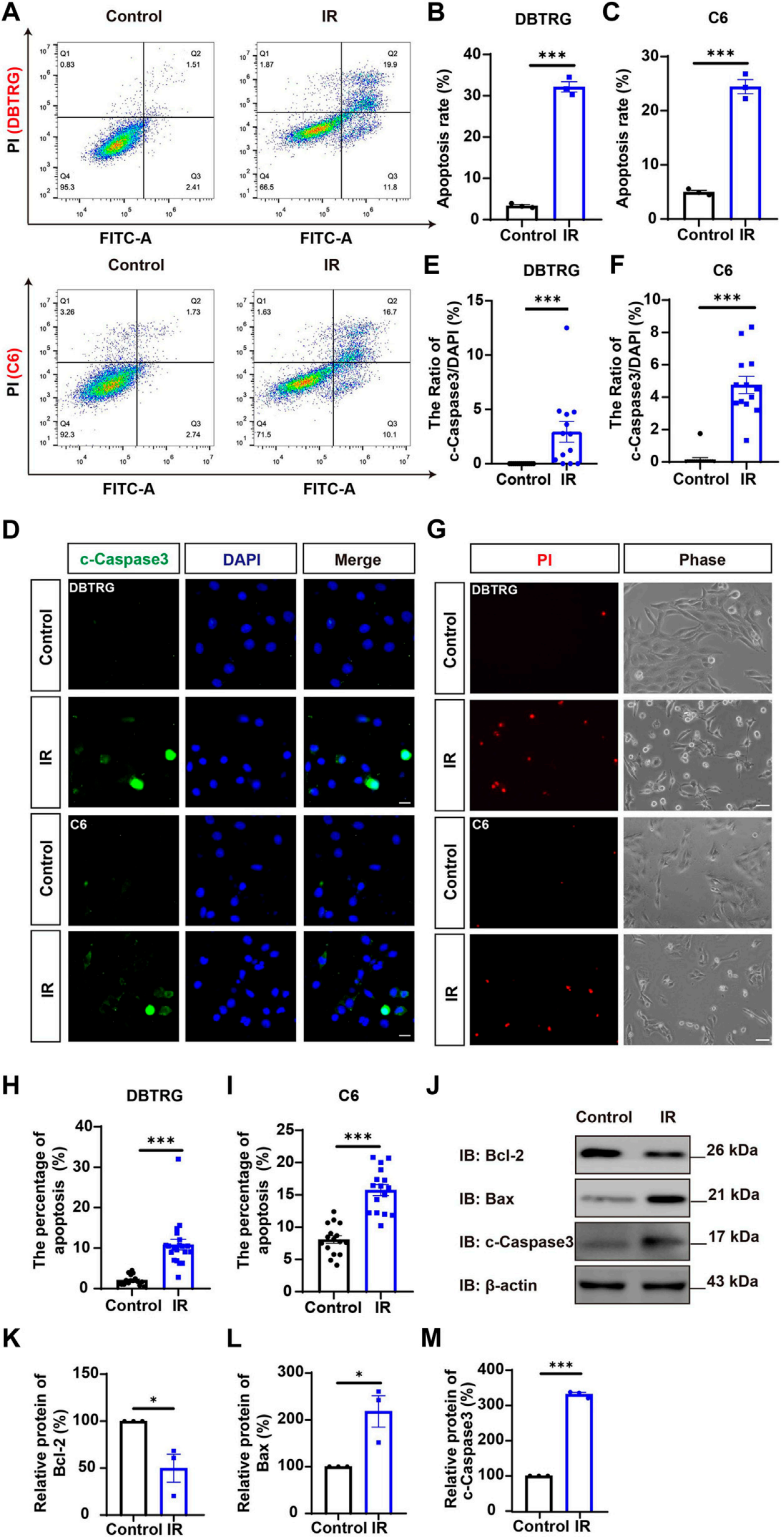
Irigenin induced the apoptosis of GBM cells. **(A)** Flow cytometry was performed to evaluate the apoptotic ratio in DBTRG and C6 cells treated with 50 μM IR and the control. **(B–C)** Quantification of the percentage of apoptotic cells in DBTRG **(B)** and C6 cells **(C)** (*n* = 3, per group). **(D)** Immunostaining analysis of cleaved-Caspase 3 (green) in DBTRG and C6 cells treated with 40 μM IR for 24 h **(E–F)** Quantitative analysis of the percentage of cleaved-Caspase 3-positive cells over total DBTRG **(E)** and C6 **(F)** cells in one field as shown in **(D)** (*n* = 13, per group). **(G)** Propidium iodide (PI) staining of DBTRG cells and C6 cells treated with 50 μM IR for 24 h **(H–I)** Quantitative analysis of the percentage of PI^+^ cells over total DBTRG **(H)** and C6 **(I)** cells as shown in **(G)** (*n* = 16, per group). **(J)** Western blot detected the expression of Bcl-2, Bax and cleaved-Caspase 3 in DBTRG cells treated with IR.** (K–M)** Quantification of the relative Bcl-2 **(K)**, Bax **(L)**, and cleaved-Caspase 3 **(M)** level as shown in **(J)** (*n* = 3, per group, normalized to control). Scale bars, 20 μm in figure **(D)** and 50 μm in figure **(G)**. Data were shown as mean ± SEM. ^*^ *p <* 0.05, ^***^ *p <* 0.001, compared with control treatment.

To determine whether the growth inhibition of irigenin is related to the induce of apoptosis, the effect on the apoptosis of irigenin was evaluated by Annexin V-FITC dual staining assay performed by flow cytometry in irigenin-treated and untreated GBM cells (Figure 3A). As shown in Figures 3A–C, the percentages of apoptotic cells in DBTRG and C6 cells treated with irigenin were significantly increased by about 10% and 16%, respectively. While there is no difference in the percentage of mechanically injured cells (Supplementary Figure S1K,L), the number of living cells decreased significantly (Supplementary Figure S1I,J). Immunostaining further confirmed that cleaved-Caspase 3^+^ cells were significantly increased in irigenin-treated DBTRG and C6 cells (Figures 3D–F). Propidium iodide (PI) staining further showed that the percentage of PI^+^ cells had a 5.5- and 2.1-fold increase both in DBTRG and C6 cells treated with irigenin for 24 h, respectively (Figures 3G–I). Furthermore, western blotting revealed that the expression levels of pro-apoptosis-related proteins cleaved-Caspase 3 and Bax were increased, whereas, the anti-apoptosis-related proteins Bcl-2 was decreased in irigenin-treated DBTRG and C6 cells (Figure 3J–M, Supplementary Figure S2C–F). These results suggested that irigenin can induce apoptosis of GBM cells.

### 2.4 Irigenin suppressed the migration of glioblastoma cells

**FIGURE 4:**
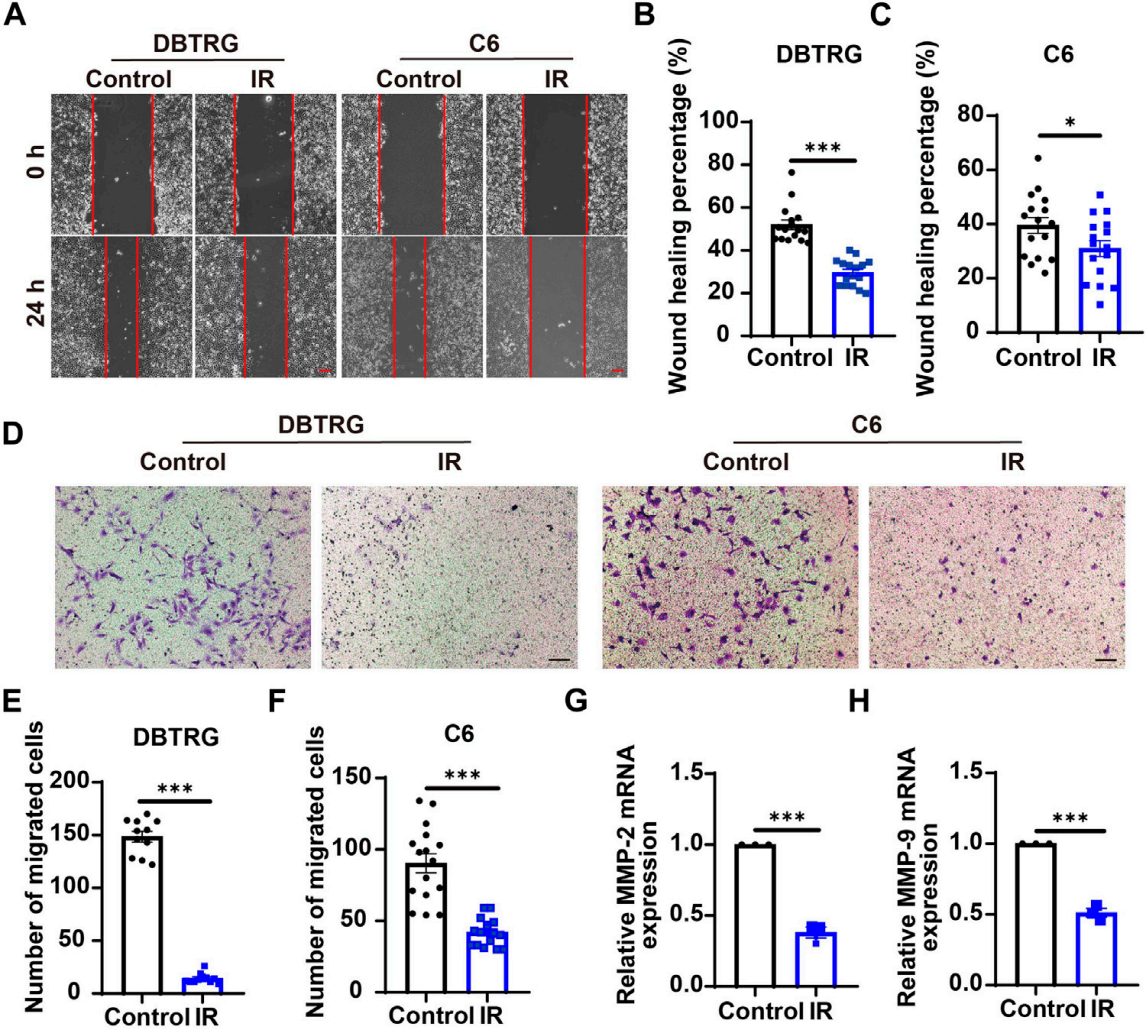
Irigenin suppressed the migration of GBM cells. **(A)** Typical images of DBTRG and C6 cells treated with 50 μM IR for 24 h in wound healing assay. **(B–C)** Quantification of wound healing index in DBTRG cells **(B)** and C6 cells **(C)** as shown in **(A)** (*n* = 16, per group). **(D)** Typical images of DBTRG and C6 cells treated with 50 μM IR for 24 h in transwell invasive assay. **(E–F)** Quantification of the number of migrated DBTRG **(E)** (*n* = 11, per group) and C6 **(F)** cells (*n* = 16, per group) as shown in **(D)**. **(G–H)** qPCR analysis results of MMP-2 mRNA levels **(G)** and MMP-9 mRNA levels **(H)** in DBTRG cells treated with or without IR (*n* = 3, per group). Scale bars, 100 μm. Data were shown as mean ± SEM. ^*^ *p <* 0.05, ^***^ *p <* 0.001, compared with control treatment.

To investigate whether irigenin can inhibit the migration of GBM cells, a wound-healing assay was performed. As shown in Figures 4A–C, irigenin treatment significantly reduced the migration of DBTRG and C6 cells after 24 h in wound healing assay. Subsequent transwell migration assay further confirmed the inhibitive effect of irigenin on GBM cell migration. Compared with the control group, irigenin treatment resulted in fewer GBM cell migration and invasion into the membrane matrix (Figures 4D–F). Consistent with these findings, we found that the mRNA expression of matrix metalloproteinase (MMP)-2 and MMP-9, which are thought to promote cancer invasion and metastasis (Zhang et al., 2017), were also significantly reduced as measured by real-time PCR analysis (Figures 4G,H). Taken together, these results suggested that irigenin can inhibit the migration of GBM cells, which may be through downregulation of MMP-2 and MMP-9 signaling.

### 2.5 Irigenin inhibited the growth of glioblastoma through suppressing YAP/β-catenin signaling pathway

**FIGURE 5:**
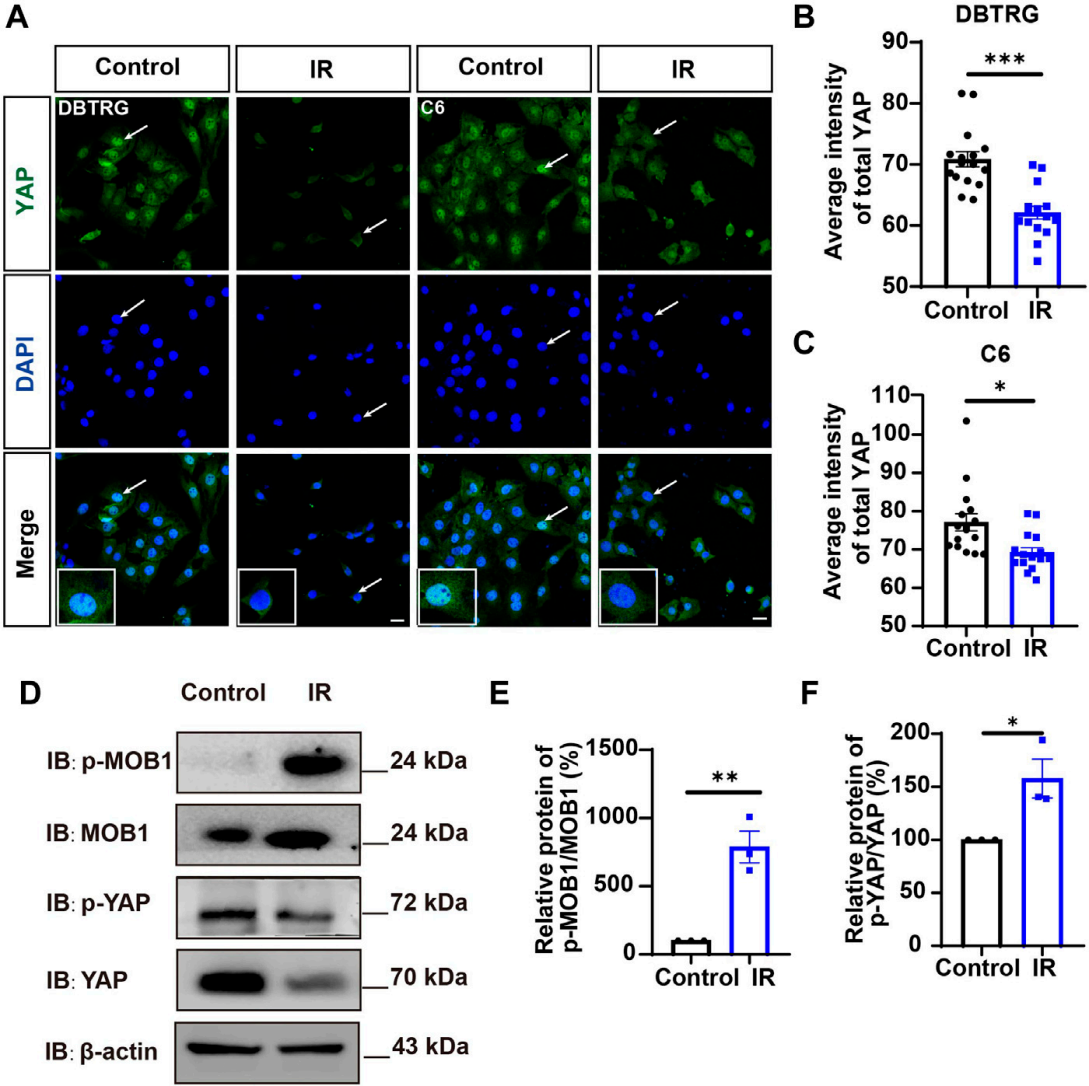
Irigenin downregulated YAP signaling in GBM cells. **(A)** Immunostaining of YAP in DBTRG and C6 cells treated with 40 μM IR. **(B–C)** Quantification of the intensity of YAP in DBTRG cells **(B)** and C6 cells **(C)** as shown in **(A)** (*n* = 16, per group). **(D)** Western blot detected the expression of p-MOB1/MOB1 and p-YAP/YAP in DBTRG cells treated with 50 μM IR. **(E–F)** Quantification of the relative level of p-MOB1/MOB1 **(E)** and p-YAP/YAP **(F)** as shown in **(D)** (*n* = 3 per group, normalized to control). Scale bars, 20 μm. Data were shown as mean ± SEM. ^*^ *p <* 0.05, ^**^ *p <* 0.01, ^***^ *p <* 0.001, compared with control treatment.

**FIGURE 6:**
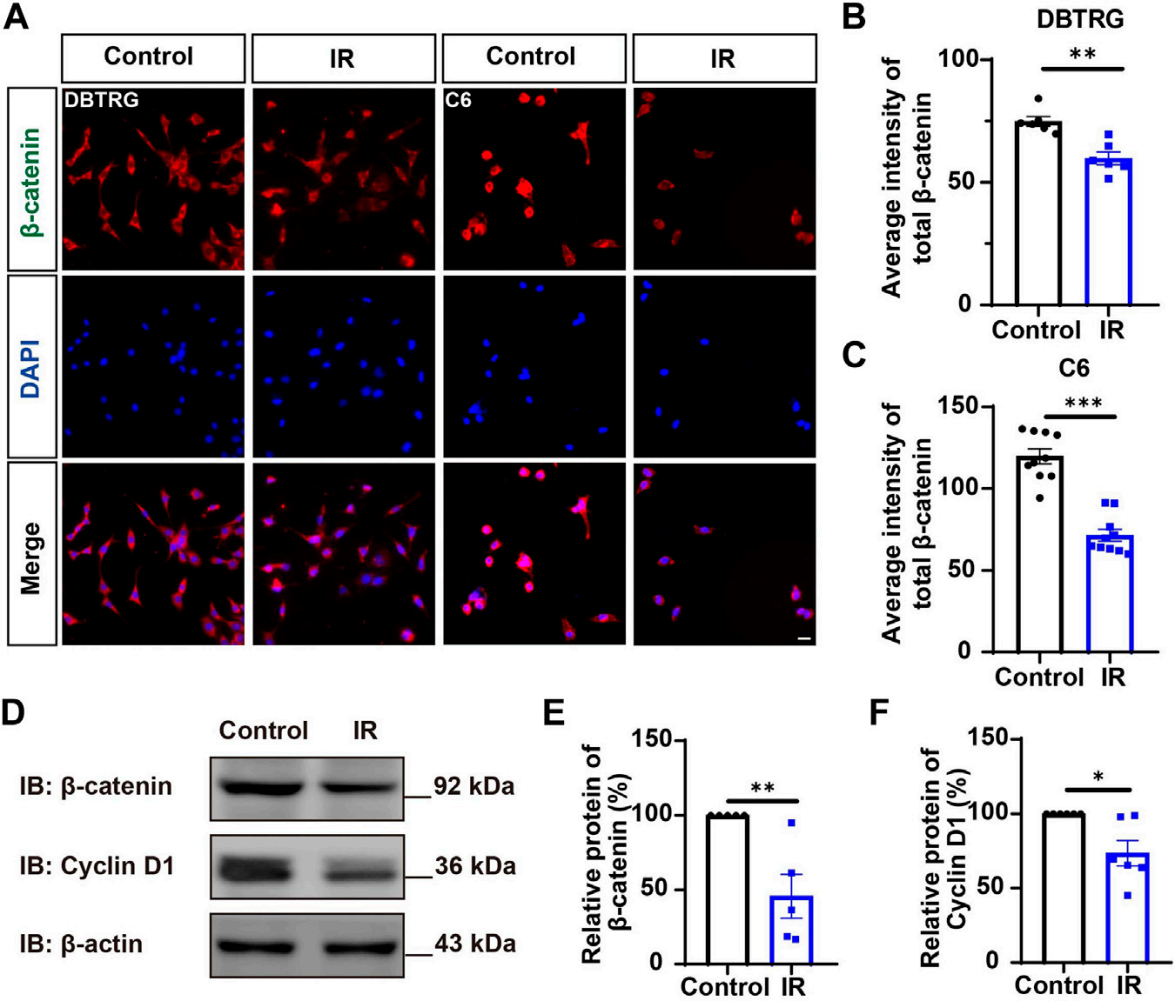
Irigenin downregulated the β-catenin signaling pathway in GBM cells. **(A)** Immunostaining of β-catenin in DBTRG and C6 cells treated with 40 μM IR. **(B–C)** Quantification of the intensity of β-catenin in DBTRG cells **(B)** and C6 cells **(C)** as shown in **(A)** (*n* = 10, per group). **(D)** Western blot detected the expression of β-catenin and Cyclin D1 in DBTRG cells treated with 50 μM IR. **(E–F)** Quantification of the relative level of β-catenin **(E)**, and Cyclin D1 **(F)** as shown in **(D)**, (*n* = 3 per group, normalized to control). Scale bars, 20 μm. Data were shown as mean ± SEM. ^*^ *p <* 0.05, ^**^ *p <* 0.01, ^***^ *p <* 0.001, compared with control treatment.

**FIGURE 7:**
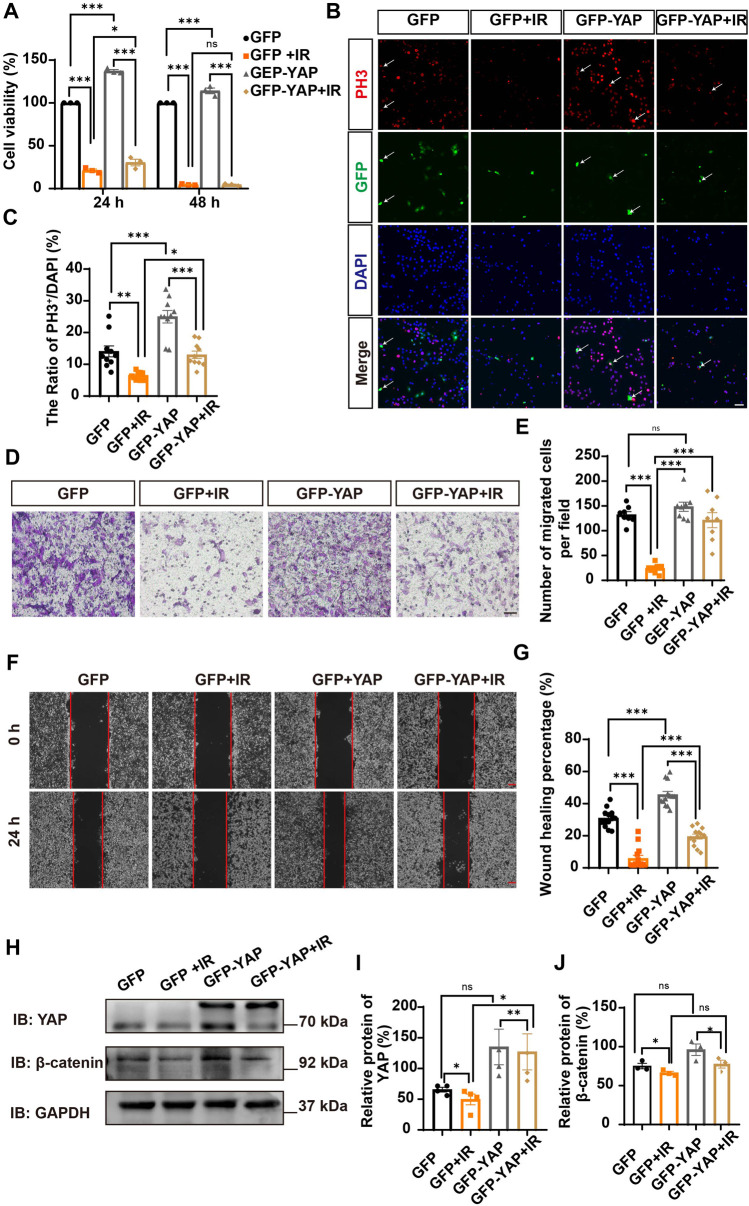
Overexpression of YAP partially rescued cell viability and proliferation inhibited by irigenin. **(A)** The cell viability by CCK-8 in C6 cells transfected with GFPN1 or GFP-YAP for 48 h and then treated with 50 μM IR and the control (*n* = 3, per group). **(B)** Immunostaining of PH3 (red) in C6 cells was transiently transfected with GFPN1 or GFP-YAP for 48 h and then treated with 40 μM IR. The white arrow indicated transfected with GFPN1 or GFP-YAP and PH3^+^ cells. **(C)** Quantitative analysis of the percentages of PH3^+^ cells over total C6 cells as shown in **(B)** (n = 10, per group). **(D)** Typical images of C6 cells transfected with GFPN1 or GFP-YAP then treated with 50 μM IR in a transwell assay. **(E)** Quantification of the number of migrated cells as shown in **(D)** (n = 8, per group). **(F)** Typical images of C6 cells transfected with GFPN1 or GFP-YAP when treated with 25 μM IR in wound healing assay. **(G)** Quantification of wound healing percentage as shown in **(F)** (*n* = 13, per group). **(H)** Western blot detected the expression of YAP and β-catenin in C6 cells transfected with GFPN1 or GFP-YAP and then treated with IR. **(I–J)** Quantification of the relative level of YAP **(I)** and β-catenin **(J)** as shown in **(H)** (n = 4/3 per group). Scale bars, 20 μm in Figure **(B)**, 100 μm in Figure **(D)** and **(F)**. Data were shown as mean ± SEM. ^*^ *p <* 0.05, ^**^ *p <* 0.01, ^***^ *p <* 0.001, compared with control treatment.

Since YAP protein plays an important role in glioma (Orr et al., 2011; Harvey et al., 2013; Zhang et al., 2018; Ouyang et al., 2020), we then examined whether irigenin inhibited growth of GBM cells through suppressing YAP signaling. We detected the expression of YAP by immunostaining experiments, and found that irigenin treatment decreased the mean fluorescence intensity of nuclear YAP (Figures 5A–C). Western blotting further showed the relative protein level of p-YAP/YAP was increased in these irigenin-treated GBM cells (Figures 5D,F; Supplementary Figure S2G, J,K), similarly, the relative level of upstream regulatory protein p-MOB1/MOB1 was also increased (Figures 5D,E; Supplementary Figure S2G–I). In addition, studies have shown that YAP1 can inhibit the activity of GSK3β, regulate the subcellular location and increase transcriptional activity of β-catenin to promote the proliferation of GBM cells (Wang et al., 2017). Next, we examined the mean fluorescence intensity of β-catenin in the control and irigenin treated cells, and found that irigenin treatment attenuated the mean fluorescence intensity of β-catenin (Figures 6A–C). Western blotting results also showed that the expression of β-catenin was down-regulated after irigenin treatment (Figures 6D,E and Supplementary Figure S2L,M). High expression of Cyclin D1 is thought to be associated with poor prognosis. Overexpressed Cyclin D1 promotes GBM proliferation, resulting in increased cell proliferation and enhanced invasive and migratory abilities (Zhao et al., 2010; Cemeli et al., 2019). As expected, we found that irigenin treatment resulted in decreased expression of Cyclin D1 (Figures 6D,F; Supplementary Figure S2L,N). These results suggested that irigenin inhibited the activity of YAP/β-catenin signaling pathway. To further confirm our results, overexpression of YAP in C6 cells was performed to investigate whether it could rescue irigenin-induced inhibition of cell proliferation, migration and apoptosis. Consistently, the results showed that overexpression of YAP indeed partially restored the decreased cell viability and slowed proliferation (Figures 7A–C), and inhibition of migration (Figures 7D–G), caused by irigenin. Meanwhile, western blotting showed that the overexpression of YAP could reverse the down-regulation of β-catenin in C6 cells by irigenin (Figures 7H–J). Taken together, these results suggested that irigenin inhibited the progression of GBM cells by inhibiting the activity of the YAP/β-catenin signaling pathway.

### 2.6 Irigenin inhibited the growth of glioblastoma cells *in vivo*

**FIGURE 8:**
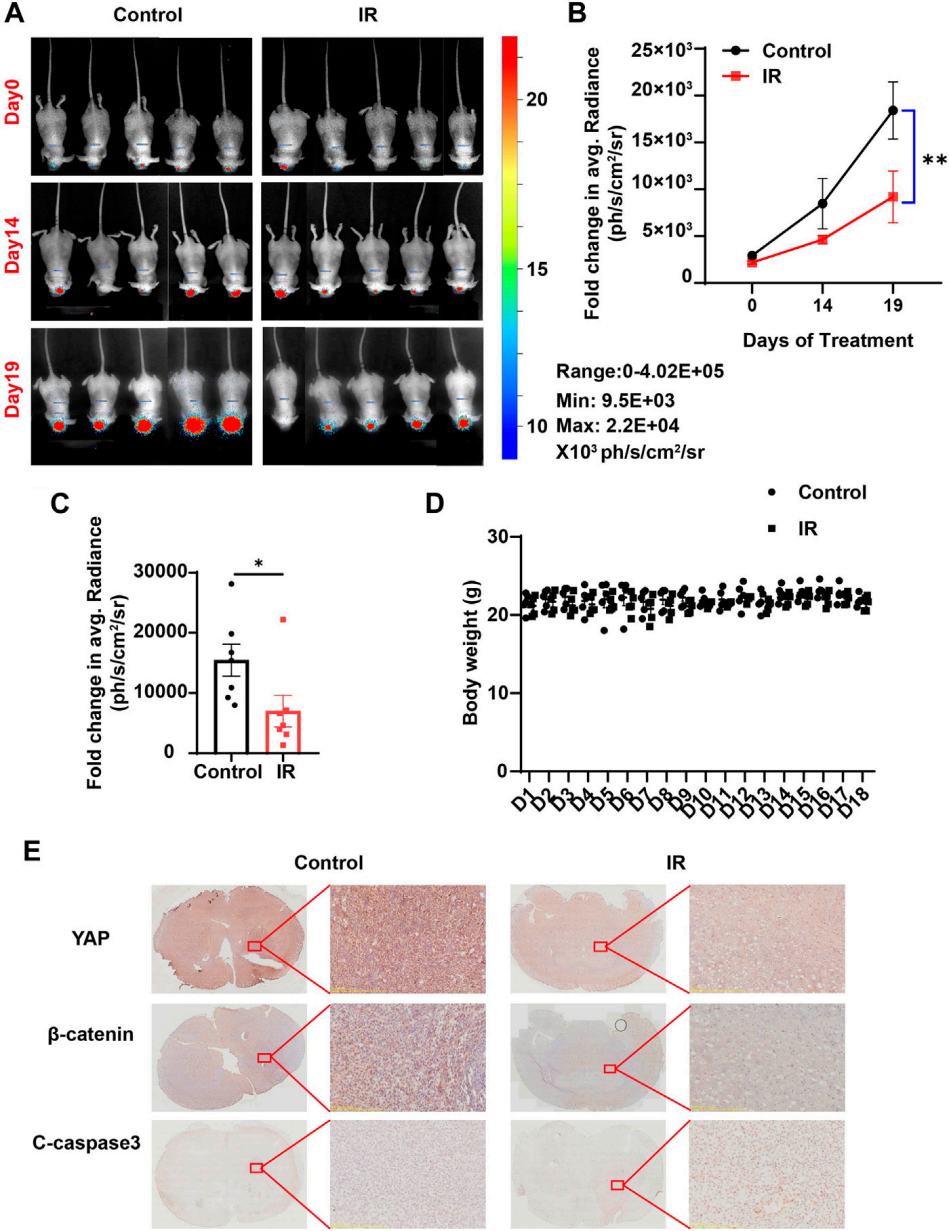
Irigenin inhibited tumorigenesis of GBM *in vivo*. **(A)** Bioluminescent imaging of disseminated C6-Luc orthotopic xenograft mice at different time points posttreatment with IR and the control group. **(B)** Fold change in the average radiance per mouse after normalization to the day 0 tumor burden as determined by bioluminescent imaging (*n* = 7 mice, per group). **(C)** Fold change in the average radiance per mouse at the experimental endpoint (day 19) for each treatment group (n = 7 mice, per group). **(D)** The body weight of the tumor-bearing mice was monitored every day (n = 5 mice, per group). **(E)** DAB staining of YAP, β-catenin and cleaved-Caspase 3 in GBM tumor of xenograft mice. Data were shown as mean ± SEM. ^*^ *p <* 0.05*,* ^**^ *p <* 0.01, compared with control treatment.

Based on the aforementioned results *in vitro*, we questioned whether irigenin also has anti-tumor effects *in vivo*. To confirm our hypothesis, we established an orthotopic transplantation model of GBM cells in nude mice. First, we injected C6 cells with the luciferase reporter gene into the striatum of BALB/c nude mice through a brain stereotaxic apparatus. After 7 d of modeling, the mice were randomly divided into two experimental groups: the control group (saline containing 10% DMSO) and the irigenin group (2 mg/ml) when the size and location of the tumor were observed by a three-dimensional bioluminescence imaging system in small animals. Mice were administrated with irigenin once a day and weighed. According to the bioluminescence fold change of C6- luciferase cells in nude mice brain, we found that compared with the control group, the treatment of irigenin significantly inhibited the growth of GBM. The fluorescence intensity value measured in the control group on the last day of administration was almost 2.2 times that of the administration group (Figures 8A–C). During the experiment, there was no significant difference in the body weight between the two groups of mice (Figure 8D). In addition, YAP and β-catenin in the administration group were significantly inhibited and cleaved-Caspase 3 was significantly activated in orthotopic model mice (Figure 8E), demonstrating that irigenin also inhibited YAP and β-catenin activities *in vivo*. Taken together, these results indicated that irigenin can inhibit GBM growth *in vivo* through inhibiting YAP/β-catenin pathway.

## 3 Discussion

**FIGURE 9:**
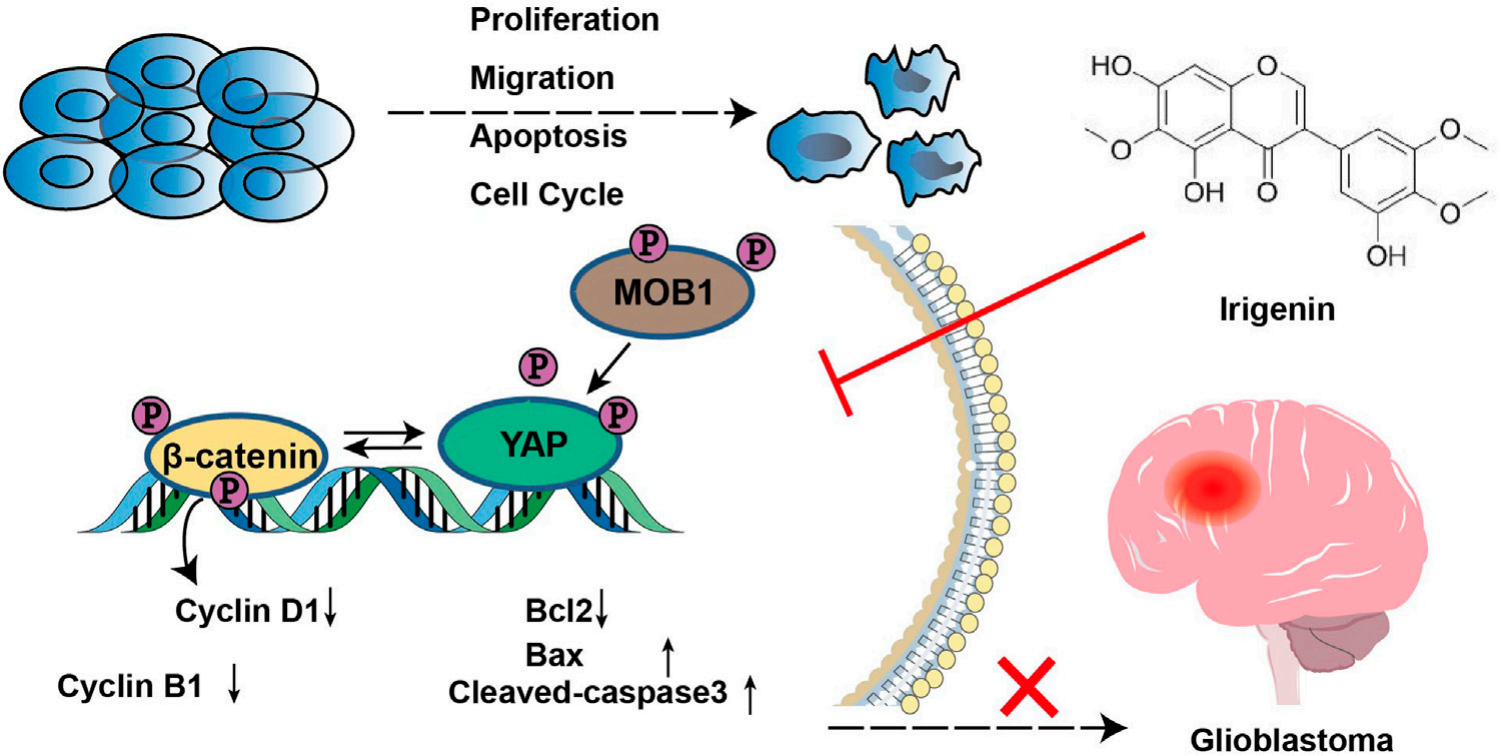
A working model of irigenin in GBM cells. Irigenin treatment inhibited the YAP/β-catenin pathway in GBM cells, which may result in inhibiting the tumorigenesis, including inhibition of cell proliferation, suppression of the migration, and induction of the apoptosis both *in vitro* and *in vivo.*

In this study, we provided evidences that irigenin inhibited the proliferation and migration of GBM cells, induced G2/M phase arrest, and regulated apoptosis-related signaling molecules to promote apoptosis by inhibiting YAP/β-catenin signaling, which suppressed the progress of GBM *in vivo* and *in vitro* progress as the working model shown (Figure 9). These findings indicated that irigenin may be an effective candidate for relieving GBM.

In fact, GBM is highly invasive and a whole-brain disease. One of the reasons for the failure of GBM therapy is that the BBB restricts the distribution of drugs in the brain. In recent years, many active ingredients of traditional Chinese medicine have made significant breakthroughs in the direction of anti-GBM, which achieved through various biological mechanisms to inhibit the occurrence of glioma (Shi et al., 2022; Wang et al., 2022). We obtained irigenin by screening on the TCMSP database (https://old.tcmsp-e.com/tcmsp.php), from which BBB value showed that it could penetrate the blood-brain barrier (Tattersall et al., 1975). It has shown that the extract of Iris (including irigenin) has a significantly better effect on the performance of AD rates in the probe test. These results suggested that irigenin can cross the BBB(Borhani et al., 2017). In mouse microglia BV-2 cells and human neuroblastoid SH-SY5Y cells, irigenin is able to resist MPP^+^-induced neurotoxicity and has neuroprotective effects (Guo et al., 2021). The potential role of irigenin as an anticancer drug in various tumor types is gradually being revealed. Irigenin can inhibit the metastasis of lung cancer cells by specifically and selectively blocking the α9β1 and α4β1 integrin binding sites on the C-C loop of the Extra Domain A (EDA) (Amin et al., 2016). In gastric cancer cells, irigenin sensitizes TNF-related apoptosis-inducing ligand (TRAIL)-induced apoptosis by enhancing the expression of pro-apoptotic molecules in the cells, as evidenced by elevated expression of Bcl-2, Caspase-3, and Caspase-8, and in order to make TRAIL produce ROS(Xu et al., 2018). Consistent with these previous studies, our *in vitro* and *in vivo* results suggested that irigenin has great potential for the treatment of this malignant glioma, inhibiting GBM cell proliferation and migration, inducing G2/M phase arrest, and promoting cell proliferation with a concentration about 50 μM.

Recent studies have shown that the occurrence and development of glioma are related to the dysregulation of various molecular mechanisms. The onset of GBM is often accompanied by mutations in genes such as TP53, EGFR, VEGF, and PTEN (Plate et al., 1992; Pore et al., 2003; Hao and Guo, 2019). Molecular signaling pathways that are known to be well studied in the carcinogenesis and progression of glioma, including Hippo/YAP, Wnt/β-catenin, STAT and Notch pathways (Zhang et al., 2012; Swiatek-Machado and Kaminska, 2013; Yan et al., 2018; Ouyang et al., 2020; ). In this study, our data showed that irigenin could inhibit the nuclear localization and expression of YAP, reduced β-catenin accumulation and suppressed the expression of its downstream protein Cyclin D1 in GBM cells.

YAP is a potential oncogene in tumor and is associated with tumor progression. Overexpression of YAP1 induces cancer cells to transform into cancer stem cells, evading cell contact inhibition, thereby enhancing their ability to invade, migrate, and proliferate (Zhao et al., 2007; Yang et al., 2013). Orr et al. found that a high level of YAP1 mRNA correlates with an aggressive molecular subset of GBM(Verhaak et al., 2010; Orr et al., 2011). In addition, the nuclear localization of YAP1 was increased in various cancer tissues and the regulation of YAP1 translocation between the cytoplasm and nucleus is a key step in hippo signaling, which is critical for physiological cell proliferation, organ size and tumor development (Huang et al., 2005; Zeng and Hong, 2008; Vlug et al., 2013; Fang et al., 2018). Furthermore, our study found that the viability of GBM cells was enhanced after YAP overexpression. This confirmed that YAP has an important role in the occurrence and maintenance of GBM, which is consistent with previous studies that YAP is able to exert its GBM-promoting ability *in vivo* and *in vitro* (Xu et al., 2010; Liu et al., 2019; ). In recent years, there have been reported crosstalk between YAP and Wnt/β-catenin signaling pathways (Orr et al., 2011; Rosenbluh et al., 2012; Park et al., 2015; Wang et al., 2017; ). β-catenin is a key downstream effector in the canonical Wnt signaling pathway, which can promote tumorigenesis and development. The β-catenin destruction complex (including GSK-3β, APC and axin) is central to the Wnt pathway. Rosenbluh et al. (Rosenbluh et al., 2012) found that in tumor cells, activated β-catenin-driven tumorigenesis is dependent on YAP1. In these tumor cells, YAP1 forms a complex with TBX5 (T-box 5, a transcription factor). When YAP1 is phosphorylated by YES1, this complex enters the nucleus and localizes to the promoters of anti-apoptotic genes, driving cancer transformation and survival. Inhibition of YAP1 phosphorylation using small-molecule inhibitors reduces tumor apoptotic capacity. Several previous studies have shown the interaction of YAP1 with β-catenin. Quinn et al. have shown that YAP and β-catenin cooperate in basal breast cancer, and immunofluorescence showed that YAP and β-catenin are colocalized in the nuclei of Wnt-Met spheres and tumors. Active YAP coimmunoprecipitated with β-catenin^GOF^ in Wnt-Met spheres. Thus, YAP and β-catenin interact in both the cytoplasm and nucleus (Quinn et al., 2021). The study of Pan et al. (2018) also has shown that β-catenin was detected in the anti-YAP, more YAP-associated β-catenin was detected in the lysates of nuclear fractions. These results thus support the view for YAP interacting with and β-catenin protein. Liu et al. (2020) found that there is a positive feedback loop between YAP and β-catenin. Wang et al. (2017) found that YAP can promote the nuclear translocation and transcriptional activity of β-catenin, indicating that YAP acts as a promoter of β-catenin rather than an inhibitor in glioma. Tang et al. (2019) demonstrated that β-catenin was significantly reduced after YAP downregulation treatment in laryngeal cancer cells. To explore the potential role in this signaling pathway, the expression levels of YAP1 and β-catenin (CTNNB1) in normal brain and GBM tissues were investigated according to the REMBRANDT database and TCGA database. As shown in Supplementary Figure S1A–D, the expressions of YAP1 and β-catenin were significantly different. According to the survival analysis based on the TGGA database, there was no correlation between YAP1 and β-catenin mRNA expression and the survival probability of GBM (Supplementary Figure S1E––G). However, the survival analysis based on the REMBRANDT revealed a correlation between YAP1 mRNA expression and the survival probability of GBM (Supplementary Figure S1F). The survival probability of GBM patients with higher YAP1 expression was significantly shorter, thus YAP1 may serve as an effective target for anticancer therapy of GBM. Our study showed that YAP overexpression partially reversed the inhibitory effect of irigenin on β-catenin protein, which indicated that irigenin acting on YAP/β-catenin can inhibit the progression of GBM, but whether it acted immediately or occurred through the modulation of YAP needs further study. In addition, we also note that irigenin can induce autophagy, which can inhibit the growth and proliferation of GBM. Irigenin causes the formation of autophagosomes in a dose-dependent manner in Coca-2 cells, and upregulates the protein expression of autophagy regulators such as Beclin-1, LC3-I, and LC3-II in a dose-dependent manner (Zhan et al., 2021). This is also the research direction that we are interested in the future, further studies are needed to fully elucidate the specific mechanisms underlying the role of irigenin in GBM progression.

In conclusion, firstly, our findings demonstrate that irigenin has anti-tumor effects on GBM cells, including inhibiting the proliferation and migration of GBM cells, triggering cell cycle arrest at the G2/M phase, and inducing cell apoptosis *in vitro*. Secondly, irigenin is effective in suppressing *in vivo* tumorigenesis. Thirdly, irigenin inhibits the GBM progress by down-regulated the YAP/β-catenin signaling. In a word, irigenin has the potential to be developed as a valid therapeutic target for GBM.

## 4 Materials and methods

### 4.1 Drugs

Irigenin (>98%) (Cas# 548–76-5) was purchased from Wuhan Zhongchang Guoyan Standard Technology Corporation. (Wuhan, China). A stock solution of 50 mM was prepared in DMSO and stored at −20°C.

### 4.2 cDNA constructs

GFP-YAP was amplified by PCR (forward primer: 5′-CGG​AAT​TCG​CCA​CCA​TGG​ATC​CCG​GGC​AGC​AG-3′, reversed primer: 5′-GGC​ACC​GGT​ACT​AAC​CAT​GTA​AGA​AAG​C-3′), cut by EcoRI and AgeI, inserted into eGFP-N1 vector, and verified by sequencing.

### 4.3 Cell culture and transfection

Glioma cell lines C6, DBTRG were kindly supplied by Prof. Maojin Yao (Sun Yat-Sen University). All cells were cultured in DMEM (Gibco) containing 10% FBS (Quacell), 1% penicillin/streptomycin (Gibco) and aseptically grown at 37°C in a humidified incubator containing 5% CO_2_. Using Lipofectamine™ 3000 Transfection Reagent (L3000-015, Invitrogen), the plasmids were transfected in C6 cells as the manufacturer’s direction. 48 h after transfection, cells were collected for subsequent experiments.

### 4.4 Cell proliferation assay

Cell viability was measured using the Cell Counting Kit-8 (CCK-8) cell counting kit (BGT-010, Biomedical Technology). Cells were resuspended, counted, and plated at approximately 4,000 cells/well in a 96-well plate, treated with increasing concentrations of irigenin (0, 10, 25, 50, 75, 100 μM) for up to 72 h. Subsequently, 10 μL of CCK-8 solution was added to each well, and the culture was continued for 2 h. The optical density of cells was measured at a wavelength of 450 nm using a microplate reader (Multiskan FC, T hermo Scientific, United States).

### 4.5 Colony-formation assays

DBTRG and C6 cells were seeded into 6-well plates at 1, 000 cells/well, then cells were treated with 50 μM irigenin after 24 h, and the cells were cultured in a 5% CO_2_ incubator at 37°C for 7 d. After washing once with 0.01 M PBS, the cells were fixed with 4% paraformaldehyde for 30 min, stained with crystal violet for 30 min and finally washed several times with PBS. Images were collected using a scanner (HP Laser MFP 131 133 135–138).

### 4.6 Transwell and wound-healing assays

For the Transwell assay, cells (2×10^4^ cells/200 μL) were placed in the upper chamber of Transwell (Corning) in serum-free medium for 36 h with 50 μM irigenin or DMSO, and then the cells were fixed and stained, and photographed and recorded using an upright fluorescence microscope (Ci-S, Nikon, Japan).

For wound healing assay, when the cell density was close to 100%, a single layer of cells was scraped with a sterile plastic tip to form a wound, and the wound gap at 0 h was recorded. Cells were then cultured in serum-free medium for 24 h with 40 μM irigenin or DMSO and photographed for recording. The reduced extent of the wound gap was calculated using Image J software.

### 4.7 Flow cytometry analysis of cell cycle and apoptosis

For cell cycle analysis, 1×10^6^ cells were collected, the cells were centrifuged after trypsinization, and washed twice with cold PBS. Following the manufacturer’s recommendations, the pelleted cells were resuspended with 500 μL DNA staining solution and 5 μL Permeabilization (Cell Cycle Staining Kit, Multi Sciences, CCS012), vortexed for 5 s, and incubated at 37°C for 30 min. DNA histograms were detected on samples using a flow cytometer (CytoFLEX S, Beckman Coulter, United States), and cell cycle distribution was analyzed using FlowJo software.

For apoptosis analysis, 1×10^6^ cells were collected, the cells were centrifuged after trypsinization, and washed twice with cold PBS. According to the manufacturer’s recommendations, the pelleted cells were resuspended in 500 μL Binding Buffer, and 3.5 μL FITC and 5 μL PI staining solution (Annexin V-FITC/PI apoptosis kit; Multi Sciences, AP101) were added for mixing and staining, followed by incubation under light for 5 min. Finally, it was determined by flow cytometry. The percentage of apoptotic cells was analyzed using FlowJo software.

### 4.8 Detection of apoptosis *via* PI staining

Glioblastoma cells were seeded in 6-well plates with 50 μM irigenin or DMSO at an appropriate density and cultured overnight. Cells were treated with a certain concentration of irigenin for 24 h. According to the instructions of the PI staining kit (Propidium Iodide, Beyotime, ST511), the working solution was prepared at 10 mg/mL, and the cells were treated with a concentration of 0.01 μg/μL. After incubation in the dark for 10 min, cells were observed and photographed with an inverted fluorescence microscope (Olympus, Japan).

### 4.9 GlioVis analysis

Glioma expression data from TCGA and REMBRANDT datasets were analyzed through the GlioVis portal (http://gliovis.bioinfo.cnio.es), a web-based program for visualizing and analyzing brain tumor expression datasets. In our work, we used GlioVis to analyze the differences in YAP1 and CTNNB1 mRNA expression levels in the normal group versus GBM, and also to analyze them for survival.

### 4.10 Western blotting

Glioblastoma cells were lysed by pre-cooled RIPA lysis buffer (P0013B; Beyotime) with protein inhibitors, lysed by sonication, and lysed on ice for 30 min. Following centrifugation at 13 000 × *g* for 15 min at 4°C. Protein was quantified with the BCA quantification kit, extracted with loading buffer, and boiled at 100°C for 10 min. Protein samples were subsequently separated using 8%~12% sodium dodecyl sulfate-polyacrylamide gel electrophoresis (SDS-PAGE) and transferred to nitrocellulose membranes (Life Sciences), blocked in TBST containing 5% skim milk for 1 h, immunoblots were incubated with primary antibodies, rabbit anti-YAP (1:1, 000, #14074, CST), rabbit anti-p-YAP (1:1, 000, #4911, CST), rabbit anti-p-MOB1 (1:500, 8699, CST), rabbit anti-MOB1 (1:500, 13730, CST), rabbit anti-β-catenin (1:500, 8480S, CST), rabbit anti-cleaved-Caspase 3 (1:1, 000, #9661, CST), rabbit anti-Cyclin B1 (1:1, 000, 1508–1, Huabio), rabbit anti-Cyclin D1 (1:1, 000, #1601–31, Huabio), rabbit anti-Bcl-2 (1:1, 000, #3498T, CST), rabbit anti-Bax (1:1, 000, ET1603-34, Huabio), rabbit anti-GAPDH (1:1, 000, 5174S, CST), and rabbit anti-β-actin (1:1, 000, 7283, Huabio) overnight at 4°C, membranes were washed three times with TBST, and incubated with horseradish peroxidase (HRP)-conjugated secondary antibodies for 1 h. After washing the membrane three times with TBST, blots were detected using the ECL detection kit (BGT-007/009, Biomedical Technology) on a GelView 6000Plus intelligent image workstation (Guangzhou Boluteng Biotechnology Co., Ltd.), and analyzed using ImageJ.

### 4.11 Immunofluorescent staining

Cells were seeded at 30, 000 cells/well in a 12-well plate with cell coverslips. The cells were treated with 50 μM irigenin or DMSO. When the cells grew to an appropriate density, the medium was changed and a certain volume of irigenin and control solvent was added, and the culture was continued for 24 h. The cells were rinsed once with PBS, fixed with 4% paraformaldehyde for 20 min, and then washed with PBS three times for 5 min each. After that, they were blocked and permeabilized with 0.1% Triton X-100 in PBS containing 5% bovine serum albumin (BSA) at room temperature for 1 h. Subsequently, cells were incubated with primary antibody overnight at 4°C, washed with PBS three times for 5 min each, and incubated with secondary antibody for 1 h at room temperature. After rinsing three more times with PBS, coverslips were mounted the slides with an anti-fluorescence decay mounting medium (S2100, Solarbio). Images were acquired by confocal (Olympus, Japan) and ImageJ software was used for analysis. The tissue sections were placed at room temperature, dried for 5~10 min, and then processed in a 60°C oven for about 30 min for antigen retrieval (2% sodium citrate solution at 92°C for 30 min). After cooling to room temperature, they were washed three times with PBS for 5 min each time. After blocked with blocking solution (5% BSA +0.3% Triton) for 1 h, Subsequently, issue sections were incubated with primary antibody overnight at 4°C, including rabbit anti-YAP (1:500, #14074, CST), rabbit anti-cleaved-Caspase 3 (1:500, #9661, CST), mouse anti-PH3 (1:2, 500, ab14955, Abcam,) and mouse anti-β-catenin (1:500, 610154, BD) antibodies, washed with PBS three times for 5 min each, and incubated with secondary antibody for 1 h at room temperature. After rinsing three more times with PBS, coverslips were mounted the slides with anti-fluorescence decay mounting medium (S2100, Solarbio). Images were acquired by confocal (FV3000, Olympus, Japan) and ImageJ software was used for analysis.

### 4.12 Immunohistochemistry

Frozen sections were baked in an oven at 60°C for 30 min, then fixed in 4% paraformaldehyde for 20 min, rinsed three times with PBS for 5 min each, and placed in a microwave oven with sodium citrate antigen retrieval solution for antigen retrieval (92°C, 30 min). Sections were then washed with PBS, permeabilized with 0.3% Triton X-100 for 30 min, and blocked with 5% bovine serum albumin (BSA) for 30 min at room temperature. Sections were then incubated overnight at 4°C with primary antibodies, including rabbit anti-YAP (1:100, #14074, CST), rabbit anti-cleaved-Caspase 3 (1:200, #9661, CST), and rabbit anti-β-catenin (1:100, 8480S, CST) antibodies. The next day, sections were rinsed three times with PBS for 5 min each and incubated with universal secondary antibodies for 30 min at 37°C. After rinsed with PBS, diaminobenzidine solution was added for signal detection. Finally, the sections were rinsed with tap water, counterstained with hematoxylin, then dehydrated with 75%, 95% and 100% alcohol and xylene, and sealed with neutral resin. Sections were photographed with an Olympus SLIDEVIEW™ VS.200 (Olympus, Germany) to obtain stained images and analyzed them.

### 4.13 RNA extraction and quantitative real-time PCR

Total RNA was extracted from DBTRG cells using RNA-easy Isolation Reagent (R701, Vazyme) according to the protocol provided by the manufacturer. RNA (100 ng) was reverse transcribed into single-stranded cDNA using HisScript^®^ II Q RT SuperMix for qPCR (+gDNA wiper) (R223-01, Vazyme) according to the manufacturer’s instructions. PCR amplification of target cDNA and internal control (β-actin) cDNA was performed using specific primers. Sample mRNA levels were quantified in a two-step method using ChamQ™ Universal SYBR^®^ qPCR Master Mix (Q711-02/03, Vazyme) on a Real-Time PCR machine (CFX Connect™ Optics Module, Bio-Rad, Singapore). Real-time PCR cycling conditions were programmed as follows: first step (95°C, 15 min), cycling step (denaturation 94°C, 15 s, annealing at 56°C or 60°C for 30 s, and final extension at 72°C for 30 s × 40 cycles). And β-actin was used as the endogenous control. Gene expression levels were expressed as ΔCt = Ct gene - Ct reference, and fold changes in gene expression were calculated by the 2^−ΔΔCt^ method. The primers used in this study were synthesized by Qingke Biotech and presented as follows: MMP-2, Forward: 5′-TCA​CTC​CTG​AGA​TCT​GCA​AAC​AG-3′, Reverse: 5′-TCA​CAG​TCC​GCC​AAA​TGA​AC-3′; MMP-9, Forward: 5′-AGT​TCC​CGG​AGT​GAG​TTG​AA-3′, Reverse: 5′-CTC​CAC​TCC​TCC​CTT​TCC​TC-3′; β-actin, Forward: 5′-AGA​CTT​CGA​GCA​GGA​GAT​GGC-3′, Reverse: 5′-TCG​TTG​CCA​ATA​GTG​ATG​ACC​TG-3′.

### 4.14 Orthotopic transplantation mouse model

The animal study was reviewed and approved by Animal Ethical and Welfare Committee of Hangzhou Normal University. Male nude mice of six-week-old BALB/c nude mice were purchased from Shanghai SLAC Laboratory Animal (Co, Ltd), and raised at the Animal Experimental Center of Hangzhou Normal University. C6 cells stably expressing Luciferase (5× 10^5^ cells per mouse) were injected into the striatum of the mice using a 69100 rotary digital stereotaxic apparatus (RWD, Shenzhen, China). To establish an *in vivo* orthotopic transplantation GBM model, were divided into two groups randomly, 7 mice in each group, 10% DMSO in saline and 20 mg/kg irigenin were administered daily by gavage per day. The body weight of the mice was measured. On the 1st, 14th, and 19th days of administration, the bioluminescence imaging of tumors was analyzed by a three-dimensional bioluminescence imaging system in small animals (PhotonIMAGER Optima, Biospace Lab, France). All nude mice were sacrificed 26 days after transplantation, and the fluorescence intensity values were analyzed to evaluate the therapeutic effect of irigenin on *in situ* GBM. The brains were collected for HE staining, DAB staining and western blotting detection.

### 4.15 Statistical analysis

All experiments were performed at least three independent times. Statistical analyses were performed using imageJ and GraphPad Prism 8.0 software. The statistical analysis significance between multiple groups was performed using analysis of variance (ANONA) and the statistical significance between two means was analyzed by student’s *t*-test. The data were expressed as the means ± standard error of the mean (SEM). Differences with *p < 0.05* were considered to be statistically significant.

## Data Availability

The original contributions presented in the study are included in the article/Supplementary Materials, further inquiries can be directed to the corresponding authors.

## Abbreviations

GBM, Glioblastoma; YAP, Yes-associated protein; qPCR, Quantitative real-time PCR; IR, irigenin.
