# Supplementary material for: Irigenin inhibits glioblastoma progression through suppressing YAP/β-catenin signaling
Source: Front Pharmacol. 2022 Nov 30;13:1027577. doi: 10.3389/fphar.2022.1027577 (PMC9748621; doi:10.3389/fphar.2022.1027577)
Supplement: Supplementary file 1 [file DataSheet1.zip › supplementary material-FS1íóFS2.docx]

**
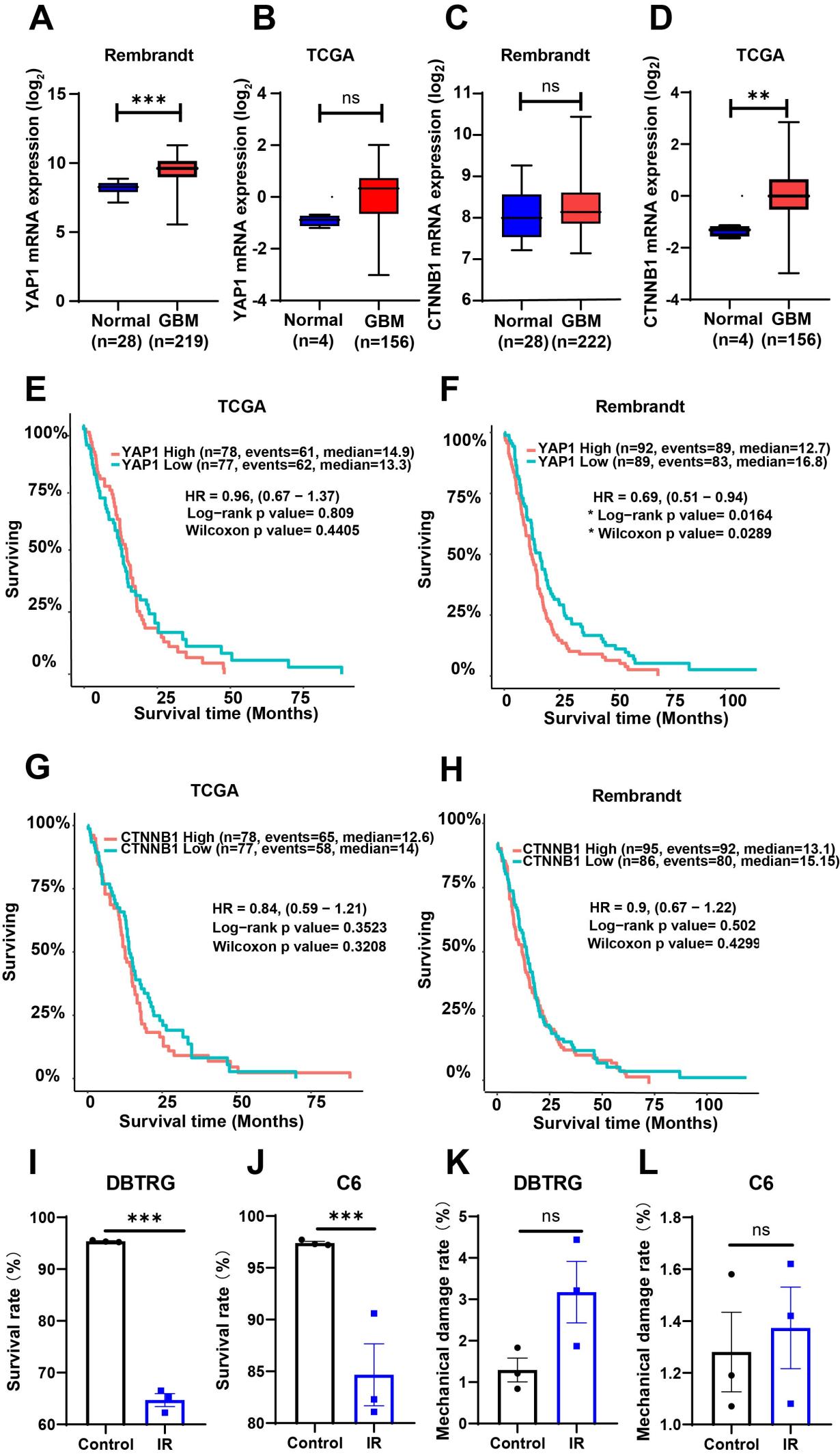
**

**Supplementary Figure 1. The YAP and β-catenin expression in GBM based on TCGA and REMBRANDT database analysis.**

(**A-D**) mRNA expression analysis of YAP1 and β-catenin (CTNNNB1) in the TCGA and REMBRANDT databases (GBM vs Normal). (**E**) Survival analysis of GBM glioma patients related to YAP1 expression according to the TCGA database. (**F**) Survival analysis of GBM glioma patients related to YAP1 expression according to the REMBRANDT database. (**G**) Survival analysis of GBM glioma patients related to β-catenin (CTNNNB1) expression according to the TCGA database. (**H**) Survival analysis of GBM glioma patients related to β-catenin (CTNNNB1) expression according to the REMBRANDT database. (**I-J**) Quantification of the percentage of living cells in DBTRG (**I**) and C6 cells (**J**) (n=3, per group). (**K-L**) Quantification of the percentage of mechanically injured cells in DBTRG (K) and C6 cells (**L**) (n=3, per group). Data were shown as mean ± SEM. *^*^p<0.05,* ^***^*p<0.001*, compared with control treatment.


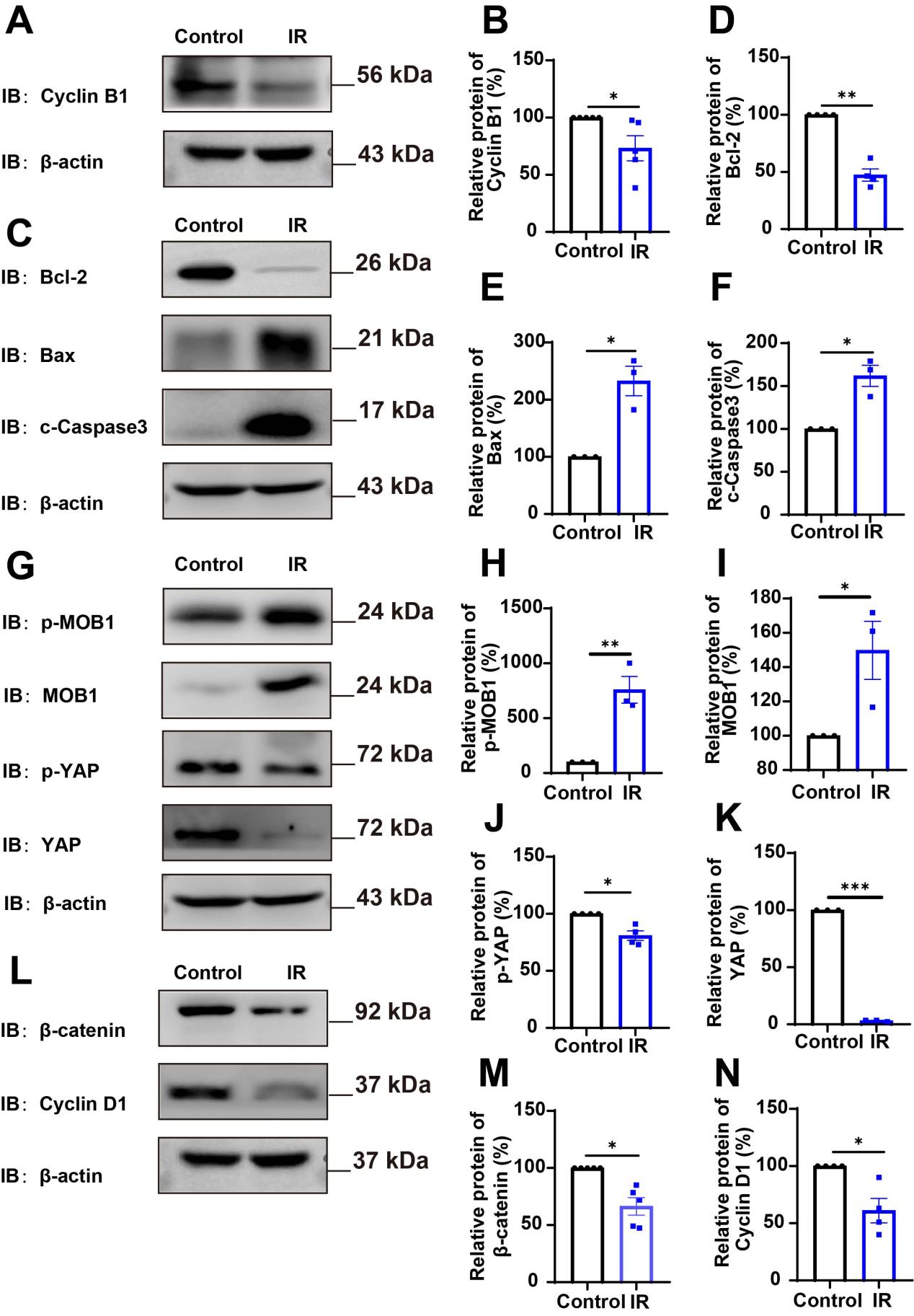


**Supplementary Figure 2. Irigenin downregulated the YAP/β-catenin signaling pathway in C6 cells.**

1. Western blot detected the expression of Cyclin B1 in C6 cells treated with 50 μM IR. (**B**) Quantification of the relative CyclinB1 level as shown in (**A**) (n=3, per group, normalized to control). (**C)** Western blot detected the expression of Bcl-2, Bax, and cleaved-Caspase3 in C6 cells treated with 50 μM IR. (**D-F**) Quantification of the relative Bcl-2 (**D**), Bax (**E**), and cleaved-Caspase 3(**F**) level as shown in (**C**) (n=3, per group, normalized to control). (**G**) Western blot detected the expression of p-MOB1, MOB1, p-YAP and YAP in C6 cells treated with 50 μM IR. (**H-I**) Quantification of the relative level of p-MOB1 (**H**), MOB1 (**I**), p-YAP (**J**), YAP (**K**) as shown in (**G**) (n=3 per group, normalized to control). (**L**) Western blot detected the expression of β-catenin and Cyclin D1 in C6 cells treated with 50 μM irigenin. (**M-N**) Quantification of the relative level of β-catenin (**M**), and Cyclin D1 (**N**) as shown in (**L**) (n=3 per group, normalized to control). Data were shown as mean ± SEM. ^*^*p<0.05*, ^**^*p<0.01*, ^***^*p<0.001*, compared with control treatment.
